# 

**Published:** 1986-06

**Authors:** 


**Contents**

53 **The History of Mental Handicap in Bristol and Bath (Part 1)**
J. Jancar
56 **Antegrade Colonic Lavage in Acute Colonic Obstruction**
Michael E. Foster and Colin D. Johnson
58 **Tranverese Myelitits and Myelography**
Peter A. Rowe and W. Peter Gorman
59 **A memorable Membershp Moment**
J. L. Burton
60 **From our Correspondents**
64 **From our Foreign Correspondent**
Edward Steen
66 **Gardening—the Great Test of Health**
T. R. Steen
68 **Surgical Club of South West England**
Abstracts of papers given at Meetings in Bristol, October 1985

